# Rehabilitation of a Patient With Maxillary Defect and Severe Attrition Using Obturator Prostheses: A Case Report

**DOI:** 10.7759/cureus.58346

**Published:** 2024-04-15

**Authors:** Grazina Fernandes, Meena Aras, Ivy Coutinho, Kennedy Mascarenhas, Praveen Rajagopal

**Affiliations:** 1 Department of Prosthodontics and Crown and Bridge, Goa Dental College and Hospital, Panaji, IND

**Keywords:** tooth wear, attrition, occlusal vertical dimension, geriatric rehabilitation, maxillofacial prosthodontics, palatal obturator, maxillary defects, full mouth rehabilitation, mucormycosis, rehabilitation

## Abstract

Surgery for palate lesions may result in oro-nasal/antral communication, which reduces a person's quality of life by affecting swallowing, speech, and food reflux. The shape and size of this obturator prosthesis might vary based on the severity of the defect. This case report describes the prosthetic rehabilitation of the patient with post-COVID mucormycosis and generalized attrition of teeth using an obturator and full mouth rehabilitation.

## Introduction

Patients with maxillary defects are more likely to experience difficulties in deglutition, mastication and can cause food reflux and hypernasal speech. Maxillary defects can be classified as acquired defects or congenital malformations. Acquired defects are a result of maxillectomy procedures for lesions of varied etiology [1,2]. Mucormycosis of the maxilla is one such lesion, which is an opportunistic fungal infection, commonly seen during the second wave of COVID-19, particularly in patients receiving corticosteroid medication and oxygen therapy [3]. It can cause severe damage to the maxilla and lungs due to its preference for blood vessels. The maxillary antrum is the primary source of infection, resulting in tissue erosion, perforation, and necrosis. It is treated with surgical debridement and resection surgery [4].

A maxillary obturator is often needed to rehabilitate a maxillary defect. The objectives of rehabilitation for total or partial maxillectomy patients include maintaining the separation of the nasal and oral cavities to enable proper deglutition and articulation, supporting the orbital contents to avoid enophthalmos and diplopia, providing support to the soft tissue to restore the midfacial contour, and achieving a satisfactory esthetic outcome [5,6].

The factors that impact the prognosis for treatment include the extent of the defect, the number and health of teeth present, the quantity of residual bony structures, and the patient's ability to adjust to the prosthesis [5,6]. The remaining natural teeth are of extreme importance as they provide retention and support to the obturator. Throughout the course of the patient's life, the occlusal surfaces of teeth may gradually wear down due to a physiological or pathological process. Tooth wear can be categorized as erosion, abrasion, attrition, or abfraction. There is a combination of these processes in several situations. Severe occlusal wear can lead to esthetic imperfections, occlusal disharmony, pulpal disease, loss of vertical dimension (VD), and decreased function [7].

In these patients, the remaining teeth need to be evaluated for wear, existing occlusal scheme, restoration, caries, and periodontal support. Any sign of tooth wear needs to be evaluated and managed carefully before fabricating obturator prosthesis. Therefore, the goal of rehabilitation of such patients is to restore function, such as speech, deglutition, mastication, and also minimizing further occlusal wear by carefully reorganizing the occlusal scheme [8].

## Case presentation

**Figure 1:**
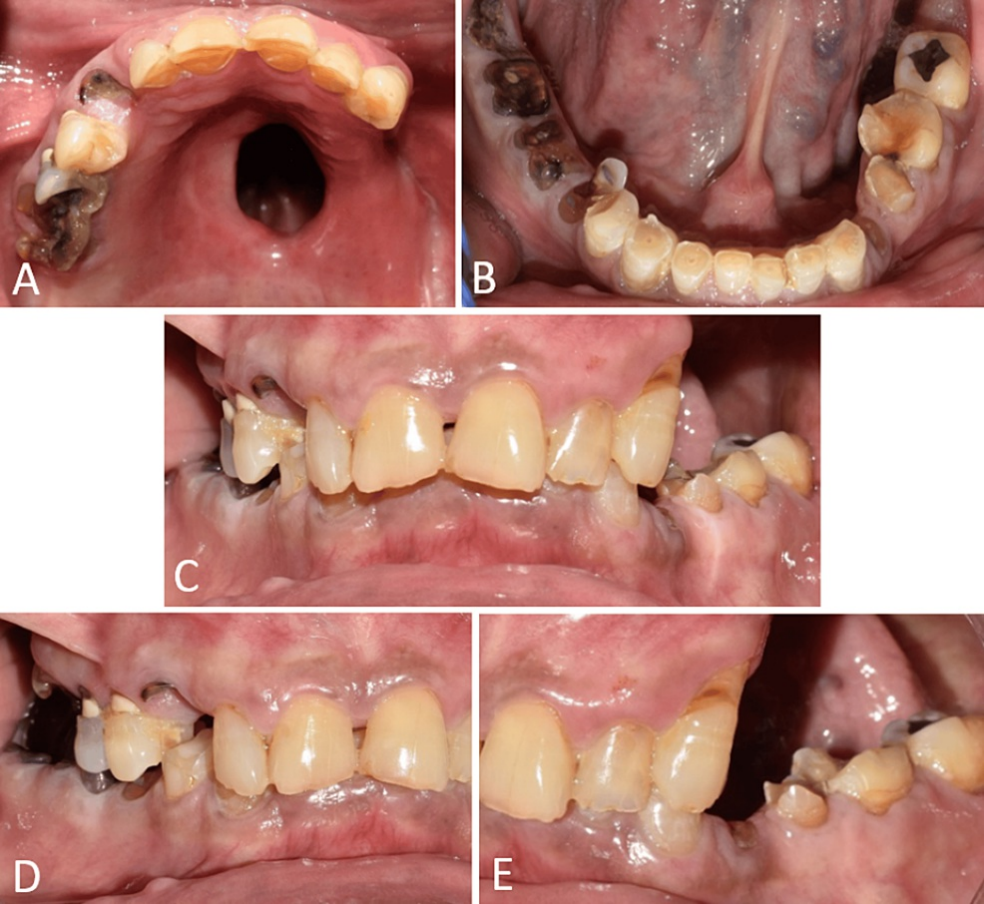
Intraoral view showing maxillary defect and severe attrition of teeth. (A) Occlusal view of the maxillary arch showing the defect in the palate; (B) occlusal view of the mandibular arch showing severe attrition of teeth; (C) frontal view on maximum intercuspation showing the severe attrition of teeth and loss of vertical dimension; (D) right lateral view showing the severe attrition and remaining roots of teeth; and (E) left lateral view showing the maxillary defect, severe attrition, and remaining roots of teeth.

**Figure 2:**
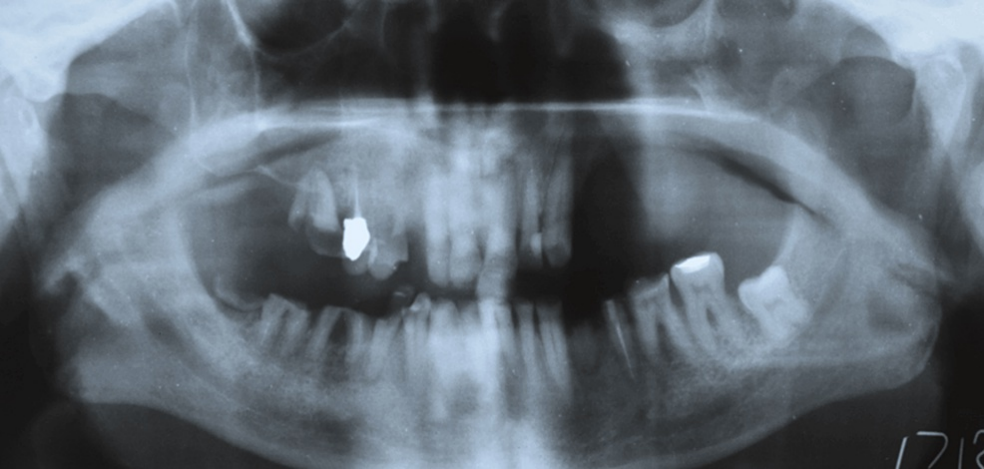
OPG showing the maxillary defect. OPG, orthopantomogram.

A 72-year-old male patient reported to the Department of Prosthodontics six months following surgery to treat mucormycosis. The main complaint was difficulty in swallowing, speech, and fluid reflux via the nasal cavity. The patient additionally reported having trouble in mastication because of tooth attrition and loss. The maxillary left buccal sulcus region, left alveolus, and a portion of the hard palate on that side were the surgical sites (Figures 1A-1E, 2). On the affected side, teeth no. 24, 25, 26, 27, and 28 were also extracted. According to Aramany's classification of maxillary defects, this defect is classified as Class II [9]. Intraoral clinical examination revealed complete healing of the surgical site, missing teeth 17, 18, 24, 25, 26, 27, and 28, and remaining roots of teeth 13, 16, 34, 45, 46, 47, and 48. The patient also had generalized attrition of teeth and loss of vertical dimension (VD) due to lack of posterior support and wear of anterior teeth.

**Figure 3:**
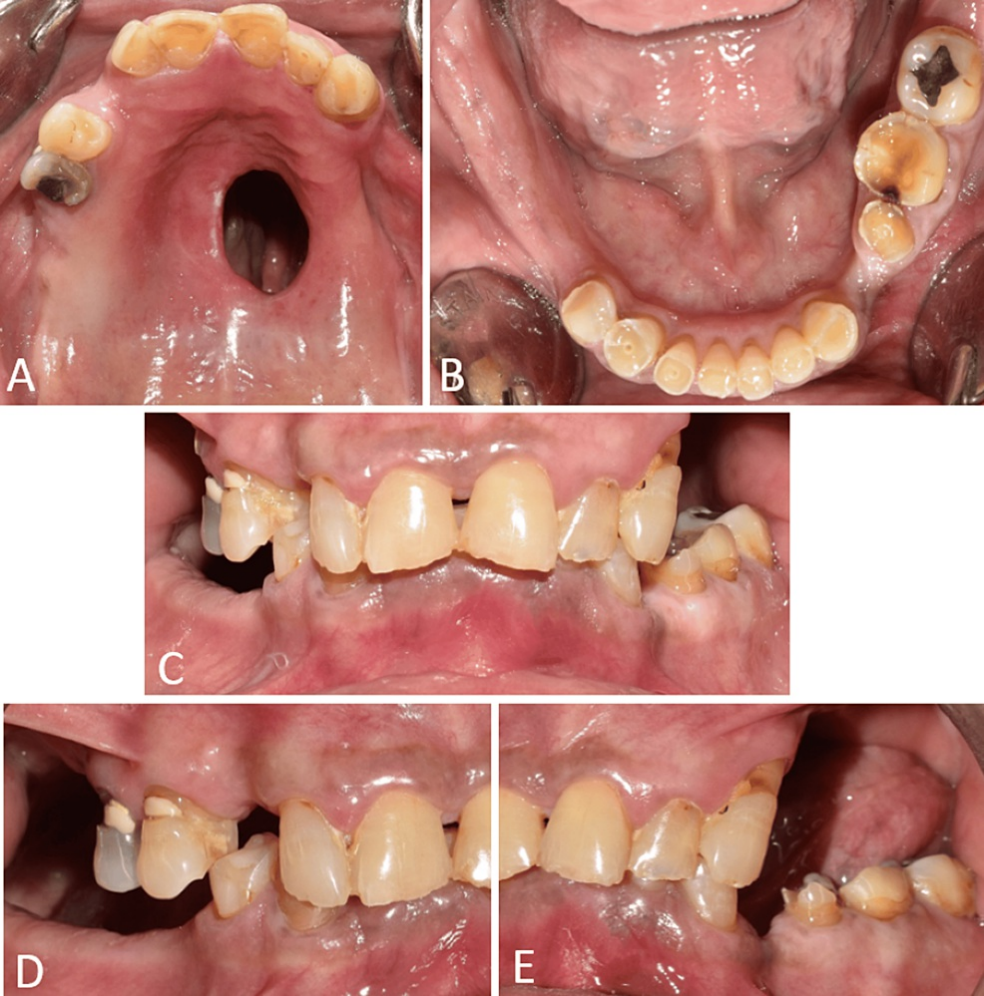
Intraoral view following extraction of remaining roots of teeth. (A) Occlusal view of the maxillary arch showing the defect in the palate; (B) occlusal view of the mandibular arch showing severe attrition of teeth; (C) frontal view on maximum intercuspation showing the severe attrition of teeth and loss of vertical dimension; (D) right lateral view showing the severe attrition of teeth; and (E) left lateral view showing the maxillary defect and severe attrition of teeth.

Diagnostic impressions were made with irreversible hydrocolloid impression material (Zhermack Tropicalgin, Zhermack SpA, Badia Polesine, Italy) followed by diagnostic mounting. A thorough treatment plan was developed based on clinical and radiological diagnosis. The treatment plan included the fabrication of a definitive obturator and full mouth rehabilitation. The remaining roots of teeth 13, 16, 34, 45, 46, 47, and 48 were extracted (Figures3A-3E).

**Figure 4:**
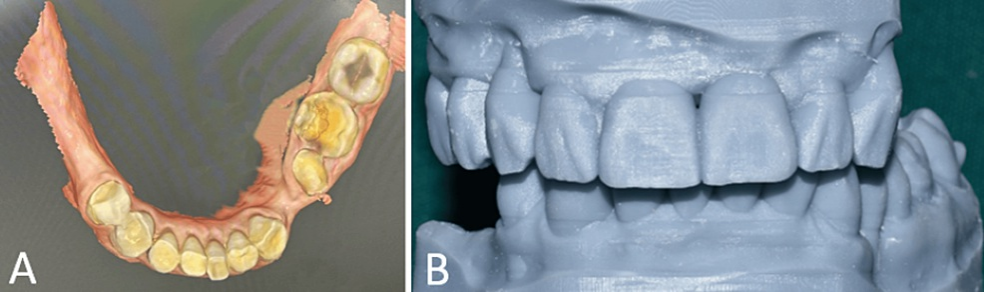
Digital phase. (A) Intraoral scan; and (B) printed models showing digital mock-up.

**Figure 5:**
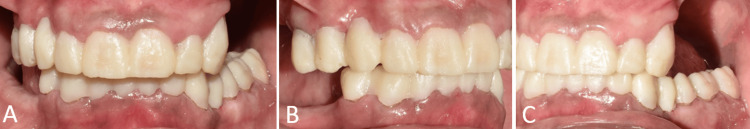
Intraoral view showing transfer of mock-up. (A) Frontal view on maximum intercuspation showing the mock-up transfer; (B) right lateral view showing the mock-up transfer; and (C) left lateral view showing the mock-up transfer.

The primary maxillary and mandibular impressions were acquired by digital impression using an intraoral scanner (irific NH100 intraoral scanner, Mumbai, India) (Figure 4A). The VD was raised using the virtual articulator based on phonetics evaluated during clinical examination. The digital mock-up (Figure 4B) was done at an increased VD and transferred to the patient using bis-acrylic temporary resin material (Care C&B Dual-Cured Temporary, Vericom, Chuncheon, Korea) (Figures 5A-5C). An interim obturator made of heat-cured acrylic resin (Stellon QC-20, Dentsply) was also given at this stage. The patient was evaluated for adaptation to the mock-up over a period of two months. There was no tenderness of the muscles of mastication and no temporomandibular joint (TMJ) discomfort.

**Figure 6:**
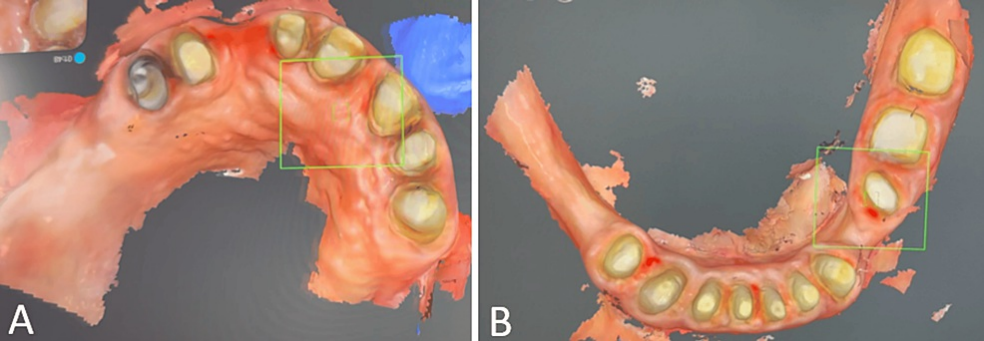
Digital scan following tooth preparation. (A) Digital scan of maxillary arch; and (B) digital scan of mandibular arch.

The teeth 11, 15, 21, 22, 23, 31, 32, 36, 37, 41, 42, 43, and 44 and 12, 14, 33, and 35 were prepared for full crowns and fixed partial denture, respectively, followed by scanning to record digital impression (Figures 6A, 6B). The interocclusal relationship was recorded using the temporary crowns.

**Figure 7:**
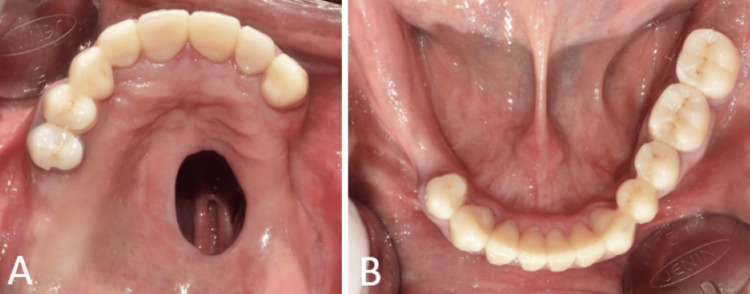
Cementation of final crowns. (A) Occlusal view of maxillary arch showing cemented crowns; and (B) occlusal view of mandibular arch showing cemented crowns.

The obturator design was planned by subjecting the scanned images to digital surveying using the software (exocad DentalCAD; exocad GmbH, Darmstadt, Germany). First, the path of insertion is automatically determined using a digital surveying tool. To determine the optimal tilt for the insertion, the software rotates the casts three dimensionally while evaluating the parallelism and undercut depth. These computations are used to automatically create the survey line. The rest seats were then incorporated into the final crowns for maxillary and mandibular cast partial denture. The final crowns (IPS e.max ZirCAD Prime, Ivoclar Vivadent, AG Schaan, Liechtenstein, Germany) were cemented (Figures 7A, 7B).

**Figure 8:**
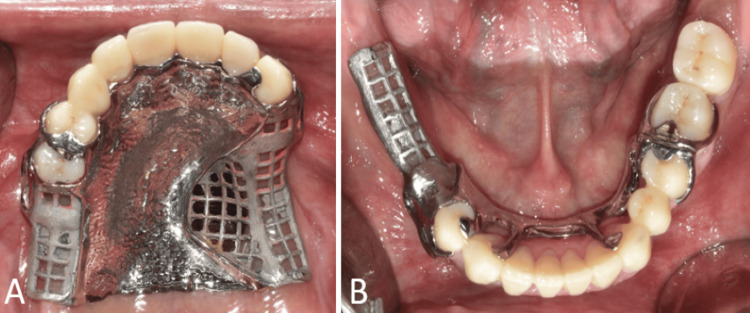
Metal framework trial. (A) Occlusal view showing framework trial of maxillary arch; and (B) occlusal view showing framework trial of mandibular arch.

The upper and lower final impressions were made using modeling compound (Green stick compound, Hiflex, Prevest DenPro, USA) and light‑body addition silicone material (Zhermack Elite HD+, Zhermack SpA, Badia Polesine, Italy) for fabricating the maxillary definitive obturator and mandibular cast partial denture. Cast metal frameworks for maxillary and mandibular arches were fabricated and checked intraorally for fit and retention (Figures 8A, 8B). Functional impressions were made with modeling compound (Green stick compound, Hiflex, Prevest DenPro, USA) and light‑body addition silicone material (Zhermack Elite HD+, Zhermack SpA, Badia Polesine, Italy) using the maxillo-mandibular frameworks, followed by pouring to fabricate the cast.

**Figure 9:**
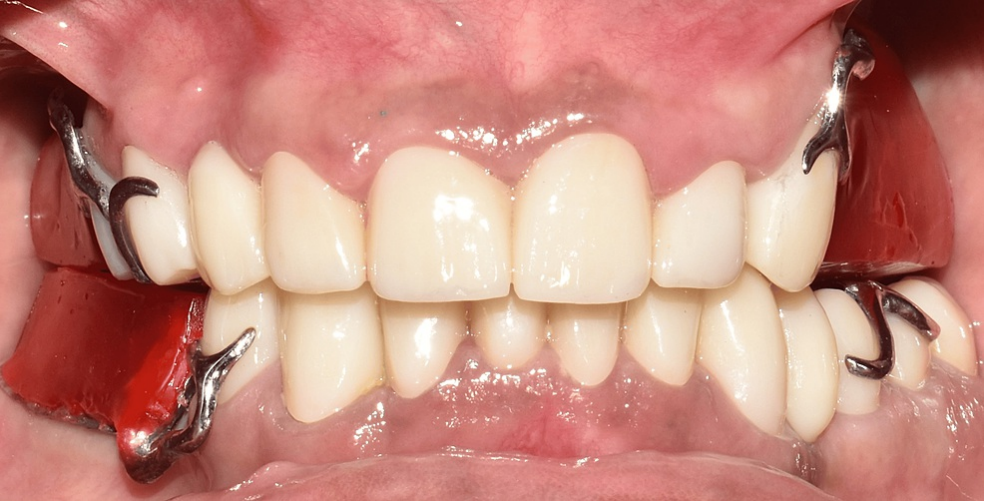
Frontal view showing maxillo-mandibular relation.

**Figure 10:**
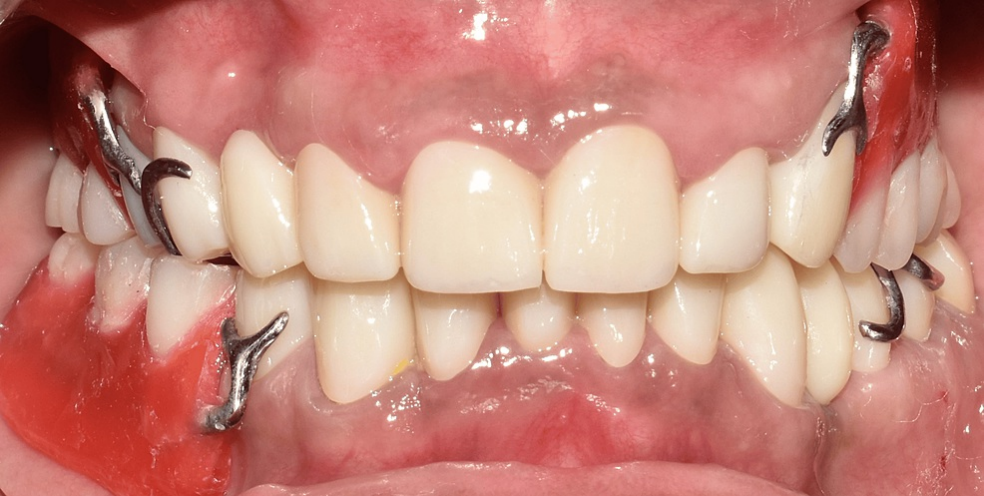
Frontal view showing try-in.

Relations were recorded (Figure 9), and after teeth arrangement, try-in (Figure 10) was done.

**Figure 11:**
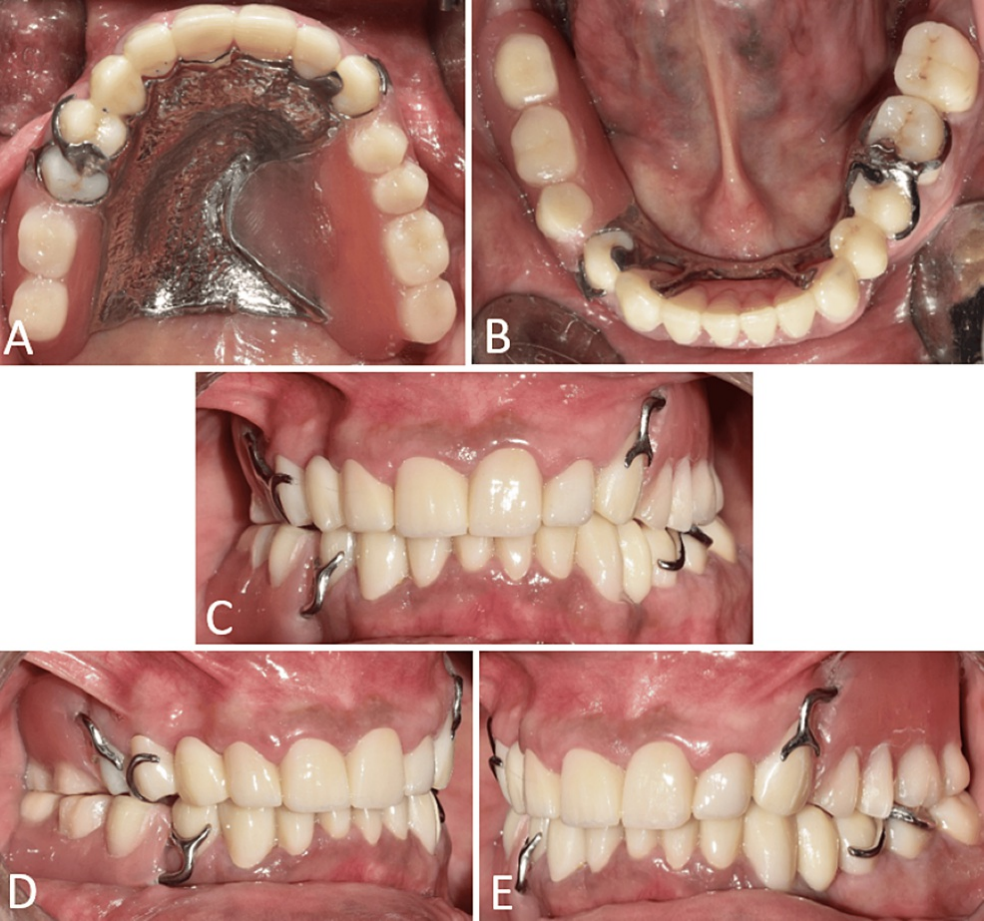
Final insertion of prosthesis. (A) Occlusal view of the maxillary arch showing the palatal obturator; (B) occlusal view of the mandibular arch showing cast partial denture; (C) frontal view on maximum intercuspation showing the final prosthesis; (D) right lateral view showing the final prosthesis; and (E) left lateral view showing the final prosthesis.

**Figure 12:**
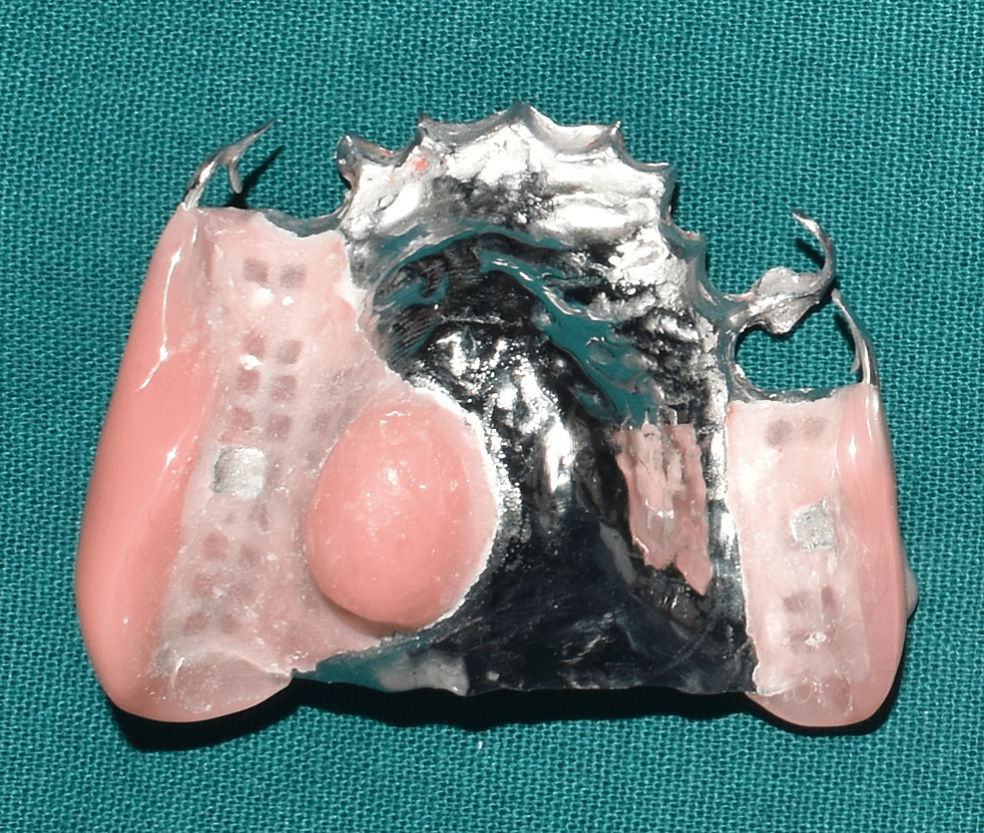
Intaglio surface of the obturator prosthesis.

**Figure 13:**
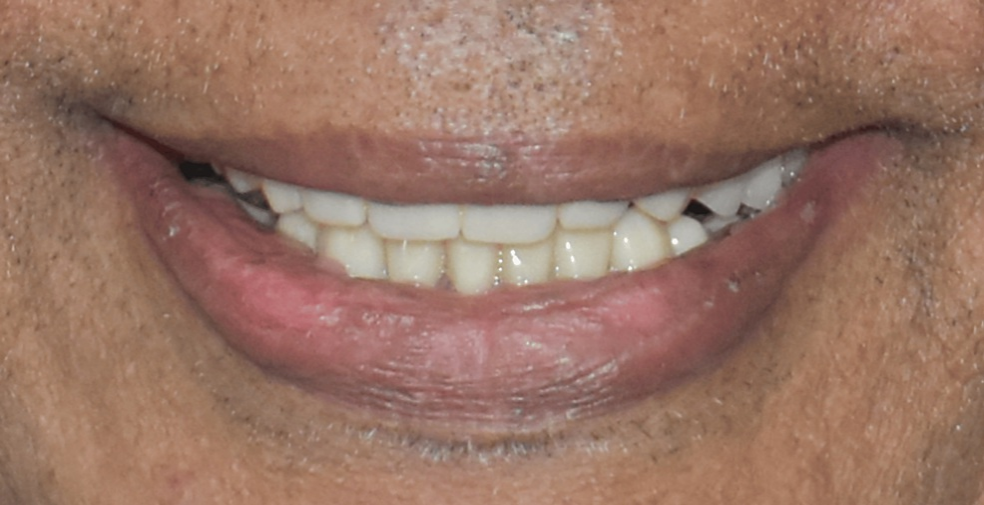
Extraoral picture of the patient after full mouth rehabilitation.

The final prosthesis was processed with heat-cured acrylic resin (SR Triplex cold, Ivoclar Vivadent AG, Schaan, Liechtenstein, Germany). The patient was given post-insertion instructions and educated regarding hygiene maintenance (Figures 11A-11E, 12, 13).

## Discussion

Prosthetic rehabilitation and surgical reconstruction are the two main treatment options available for such defects. Surgical flap reconstruction entails longer surgical time, a greater demand for skilled professionals, a potential risk of donor morbidity at the flap harvest location, and chances of recurrence in cases such as mucormycosis. The prosthetic rehabilitation includes fixed implant-supported or removable obturator prosthesis. The preferred treatment for enhanced function is zygomatic and pterygoid implants. However, there are many drawbacks, including expense, a second surgery, a longer recovery period, and challenges with prosthesis care [10].

An alternative approach to treatment is the removable obturator, which offers adequate obturation of the defect and easy prosthesis maintenance. An interdisciplinary approach should be followed which includes discussion with the surgeon and preservation of teeth of strategic importance. In planned cases, three forms of obturators can be given such as immediate (before 48 hours), intermediate (between 48 hours and three months), and definitive (after three months). The prosthesis for the definitive obturator may consist of a single piece or two pieces [11].

Digital scanning is applied in prosthodontics and restorative dental treatments due to the rapid development of computer-aided design/computer-assisted manufacturing technologies. When compared to the conventional impression method, digital impressions have several benefits. These include the ability to save time and resources, the avoidance of dental material distortion during cast fabrication, the ability to store and transfer scanned images conveniently, and the prevention of patient discomfort due to the absence of actual impression material [12].

Managing worn dentition with either fixed or removable prosthesis is a complex scenario that is very challenging to restore. Evaluation of the vertical dimension is crucial for management, and each case requires a meticulously detailed treatment plan. The clinical evaluation of a patient wearing a temporary prosthesis or diagnostic splint confirms the patient's tolerance to alterations in the vertical dimension of occlusion [13]. Rehabilitation with posterior support provided by removable dentures and anterior full crowns is common and affordable. If the patient fails to wear the denture or residual ridge resorption progresses, the restored anterior teeth may be subjected to high occlusal stresses. It is crucial to have regular checkups for adjustments [14].

## Conclusions

Satisfactory results can be achieved with a correct diagnosis and a well-planned treatment strategy. Obturator prosthesis rehabilitation is a functional and effective treatment option. This case report describes the prosthetic rehabilitation of severely worn dentition and acquired maxillary defect with one piece definitive obturator. The prosthesis enhanced the ability to swallow and speak. The rehabilitation had a good psychological impact on the patient.
